# AMPK activation improves recovery from pneumonia-induced lung injury via reduction of er-stress and apoptosis in alveolar epithelial cells

**DOI:** 10.1186/s12931-023-02483-6

**Published:** 2023-07-12

**Authors:** Eugene Becker, Maroof Husain, Nathaniel Bone, Samuel Smith, Peter Morris, Jaroslaw W. Zmijewski

**Affiliations:** Division of Pulmonary, Allergy and Critical Care, Department of Medicine, University of Alabama at Birmingham, 901 19th St. South BMRII 406, Birmingham, AL 35294-0012 USA

**Keywords:** Bacterial infections, Pneumonia, Endoplasmic reticulum, Mitochondria, Alveolar epithelial cells, Efferocytosis

## Abstract

**Background:**

Bacterial pneumonia and related lung injury are among the most frequent causes of mortality in intensive care units, but also inflict serious and prolonged respiratory complications among survivors. Given that endoplasmic reticulum (ER) stress is a hallmark of sepsis-related alveolar epithelial cell (AEC) dysfunction, we tested if AMP-activated protein kinase (AMPK) affects recovery from ER stress and apoptosis of AECs during post-bacterial infection.

**Methods:**

In a murine model of lung injury by *P. aeruginosa* non-lethal infection, therapeutic interventions included AMPK activator metformin or GSK-3β inhibitor Tideglusib for 96 h. Recovery from AEC injury was evidenced by accumulation of soluble T-1α (AEC Type 1 marker) in BAL fluids along with fluorescence analysis of ER-stress (CHOP) and apoptosis (TUNEL) in lung sections. AMPK phosphorylation status and mediators of ER stress were determined via Immunoblot analysis from lung homogenates. Macrophage-dependent clearance of apoptotic cells was determined using flow cytometry assay.

**Results:**

*P. aeruginosa*-induced lung injury resulted in accumulation of neutrophils and cellular debris in the alveolar space along with persistent (96 h) ER-stress and apoptosis of AECs. While lung infection triggered AMPK inactivation (de-phosphorylation of Thr172-AMPK), metformin and Tideglusib promptly restored the AMPK activation status. In post infected mice, AMPK activation reduced indices of lung injury, ER stress and related apoptosis of AECs, as early as 24 h post administration of AMPK activators. In addition, we demonstrate that the extent of apoptotic cell accumulation is also dependent on AMPK-mediated clearance of apoptotic cells by macrophages.

**Conclusions:**

Our study provides important insights into AMPK function in the preservation of AEC viability after bacterial infection, in particular due reduction of ER-stress and apoptosis, thereby promoting effective recovery from lung injury after pneumonia.

**Supplementary Information:**

The online version contains supplementary material available at 10.1186/s12931-023-02483-6.

## Background

Pneumonia is a leading cause of in hospital morbidity and mortality, affecting critically ill patients worldwide [1, 2]. Community acquired pneumonia, nosocomial infections and mechanical ventilation-related pneumonias are frequent causes of acute respiratory distress syndrome (ARDS). ARDS can also develop after various insults including non-pulmonary sepsis, including trauma and severe blood loss. The development of lung injury is a complex event, including accumulation of activated innate immune cells, i.e., neutrophils with exaggerated pro-inflammatory activation, as well as alveolar and epithelial dysfunction and apoptosis [3, 4]. This is accompanied by an adverse endothelial barrier permeabilization and coagulation, followed by the onset of edema and respiratory failure. Prompt and appropriate diagnosis and treatment of bacterial pathogens in conjunction with ventilator support and fluid resuscitation comprises initial treatment. However, these supportive interventions are often ineffective due to diminished response to fluids and the presence of antibiotic resistant pathogens. It is important to note that most patients survive severe infections/sepsis; however, within the same hospitalization, many develop post-septic complications, including life-threatening decline of immune function (immunosuppression) often associated with subsequent lung or non-pulmonary infections [1, 5, 6]. In addition, apoptosis of alveolar epithelial cells and subsequent remodeling are frequently implicated in post-ARDS interstitial fibrosis, inflicting prolong adverse impact on respiratory function [7–9]. Despite significant progress in understanding pathophysiological conditions involved in the development severe lung infections and injury, effective therapeutics are not available for critically ill patients that enhance the recovery process among ARDS survivors. Notably, although many pre-emptive approaches have been developed in animal models, the clinical trials for sepsis and ARDS have mostly disappointing outcomes [6, 10–12].

The endoplasmic reticulum (ER) plays a fundamental role in cellular homeostasis, given its direct impact on protein folding, synthesis of lipids, sterols and calcium storage [13]. ER stress and resulting unfolded protein response (UPR) occur in a range of pulmonary insults and play important roles in many respiratory disorders, including cystic fibrosis, asthma and COPD [14, 15]. In particular, ER stress is a hallmark of sepsis-induced lung epithelial injury and apoptosis, including in pneumonias triggered by *Pseudomonas, Legionella* and *Aspergillus* [16]. ER-stress and mitochondrial dysfunction parallel the development of organ injury in ARDS and correlate with severity of lung injury and mortality [17, 18]. Recent studies, including our previous results in polymicrobial sepsis and hemorrhage models, have demonstrated that modulation of metabolic and bioenergetic functions in lung epithelial, endothelial and immune cells reduces the severity of acute lung injury (ALI) [19–22]. However, less is known about therapeutic interventions that target bioenergetics and closely related proteostasis to improve recovery from injury. This is an important question, given that many critically ill patients progress experience a prolonged hospitalization with high associated mortality and for survivors there is a prolonged recovery following hospital discharge [1, 5].

The previous studies, including our results, have demonstrated that AMP-activated protein kinase (AMPK) has a tremendous protective impact against the development of lung injury [20, 22–25], and also accelerated the resolution of lung fibrosis [26–29]. AMPK is a serine/threonine protein kinase with a unique mechanisms of activation associated with metabolic stress due to depletion of ATP and accumulation of ADP and AMP, e.g., when oxygen and nutrients bioavailability is limited [30–34]. Within α/β/γ subunits of AMPK heterotrimeric complex, AMP/ADP-dependent stimulation of γ subunit allows for allosteric domain rearrangement of the catalytic AMPKα domain to be phosphorylated by upstream kinases, for maximal activation. Once activated, AMPK regulates the major metabolic pathways, including carbohydrate, lipid and protein synthesis. AMPK activity is essential for mitochondrial bioenergetic function, including mitochondrial biogenesis, regulation of dynamics and quality control [19, 34–36].

We hypothesize that AMPK activation is a feasible therapeutic target for recovery from mitochondrial-ER stress after severe lung bacterial infections. This is a plausible concept, given that inflammatory conditions impair crosstalk between mitochondrial-ER and suppress AMPK activity i.e. sepsis [37, 38]. Recently, we have shown that Glycogen synthase kinase 3β (GSK-3β) inhibits AMPK in pre-clinical models of sepsis [20]. However, whether AMPK activation promotes the recovery from ER stress after bacterial pneumonia, remain to be determined. To test this possibility, we implemented therapeutic intervention with metformin to activate AMPK in a mouse model of bacterial pneumonia. The second intervention included GSK-3β inhibitor Tideglusib, to diminish GSK-3β-mediated suppression of AMPK.

## Methods

### Mice

All experiments were conducted in accordance with approved protocols by the University of Alabama at Birmingham Institutional Animal Care and Use Committee. Male C57BL/6 mice were purchased from The Jackson Laboratory (Bar Harbor, ME). Mice were given food and water ad libitum and kept on a 12-h light–dark cycle. Mice 10 to 12 weeks of age were used for bacterial lung infections. The animal experiments performed for this study were carried out in compliance with the ARRIVE guidelines.

### ***P. aeruginosa***-induced pneumonia in mice

*Pseudomonas aeruginosa* deposition into the mouse pharynx followed by aspiration was conducted using previously described methods. In brief, mice anesthetized with isoflurane were suspended by their upper incisors on a 60° incline board, tongue was gently extended followed by oropharyngeal deposition of PBS alone (control; 50 µl) or *P. aeruginosa*, wild-type strain K (PAK; 2.5 × 10^6^/mouse) in PBS (50 µl). Mice received metformin (65 mg/kg; i.p.) or Tideglusib (50 mg/kg; i.p.) 24 h post infections, once a day, every other day. Lung sections and homogenates were prepared from control (vehicle) group and 24, 48, 72 and 96 h post PAK instillation.

### Reagents and antibodies

Metformin, RPMI 1640 media are obtained from Sigma-Aldrich (St. Louis, MO). Antibodies for phospho-Thr172-AMPK, AMPKα1, phosphor-Ser51-eIF2α and HO-1 are from Cell Signaling Technology (Beverly, MA). CHOP and β-actin are purchased from Santa Cruz Biotechnology (Dallas, TX), whereas anti-T-1α IgG and IL-1, MIP-2 and IgM ELISA kits are from R&D Systems (Minneapolis, MN). Horse Radish Peroxidase-conjugated antibodies are obtained from Bio-Rad (Hercules, CA), whereas emulsion oil solution containing 4′,6-diamidino-2-phenylindole (DAPI) is from Vector Laboratories (Burlingame, CA).

### Lung histology and imaging

Lung section preparation, lung injury score, H&E and fluorescence staining were conducted using methods previously established in our laboratory [19, 26]. In brief, lungs were inflated with 1 ml paraformaldehyde in PBS (4%) and embedded with paraffin. Prior to staining, lung Sect. (5-µm-thick) were deparaffinized in serial solutions of Citrisolv (Fisher Scientific, Pittsburgh, PA), isopropyl alcohol, and water, followed by H&E staining (Vector labs) in selected indirect immunofluorescence staining antigen retrieval via steaming in citric acid (10 mM, pH 6.0) for 20 min and cooling for additional 20 min. Next, lung sections washed with PBS and blocked with BSA (3%) for 90 min were incubated with anti-CHOP and anti-T-1α antibody overnight, at 4 °C. Secondary Donkey anti Chicken Alexa 594 or goat Alexa 488 labeled antibody were added for 60 min. Nuclei were stained using the emulsion oil solution containing DAPI. Fluorescence intensity was measured in randomly chosen areas of lung sections from control and post-PAK mice, including mice that were treated with metformin or Tideglusib. Images were acquired using a fluorescent microscope Keyence BZx10. The levels of fluorescence in randomly selected areas were quantitated and displayed as two-dimensional scattergrams using HCImage, Hamamatsu’s image acquisition and analysis software.

### Cytokine ELISA

ELISA was used to measure cytokine levels in bronchoalveolar lavage (BAL) fluids as previously described. Levels of TNF-α, MIP-2, IL-1 and IgM were determined using ELISA kits according to manufacturer’s instructions by R&D Systems (Minneapolis, MN).

### Western blot analysis

The Western blots and quantitative analysis of AMPK phosphorylation, T-1α, eIF-α, HO-1 and CHOP were performed as described in our previous studies [19, 20]. For WB analysis of T-1α in BALs, each loaded sample was prepared using 30 µl of lavages.

### Protein concentration and cell counts in BAL fluids

The protein concentration in BAL fluids was determined by Bradford assay with Bio-Rad protein assay dye reagent concentrate (Bio-Rad, 500-0006). Total cell counts in BAL fluids were determined by hematocytometer counts by light microscopy.

### Efferocytosis assay

Efferocytosis assay was performed using (fluorescently labeled with T-1α) murine apoptotic L2, alveolar cell line (ATCC) and peritoneal mature macrophages, as previously conducted in our laboratory [39]. Apoptosis of L2 cell was induced upon exposure to UV for 10 min, incubated for 3 h, and then the extent of apoptosis determined using Flow cytometry in conjunction with Annexin V staining.

### Statistical analysis

One-way ANOVA with Tukey’s post hoc test was used to determine the statistical significance among multi groups, with normal distribution. For two groups, statistical significance was established using Student’s *t*-test. These analyses are performed with Microsoft Excel and Prism GraphPad (version 8.4.2). A *P* value < 0.05 is considered significant.

## Results

### Therapeutic interventions with metformin and Tideglusib activate AMPK and promote resolution of lung injury after ***P. aeruginosa***-induced lung injury

**Fig. 1:**
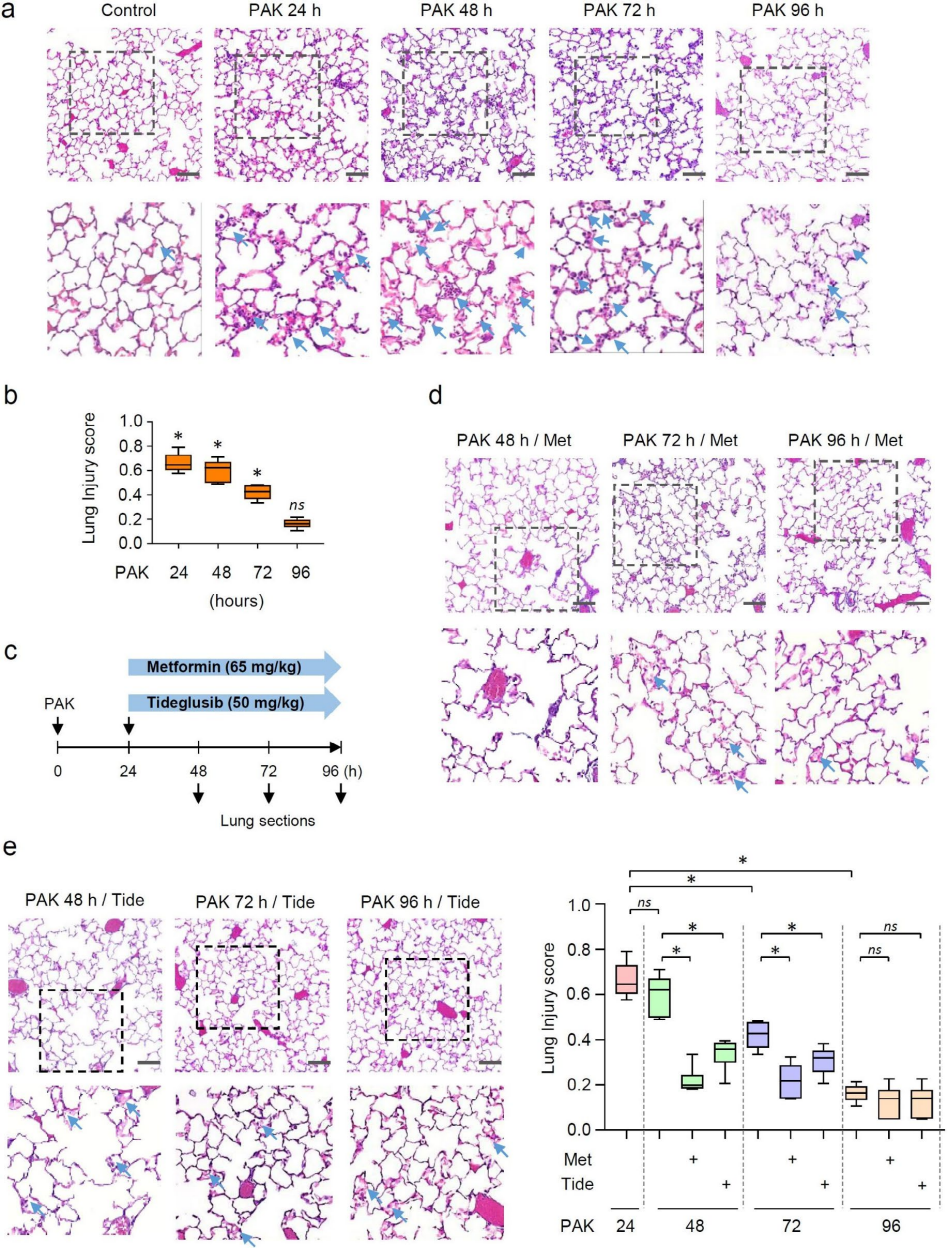
AMPK activation improved recovery from lung injury in mice subjected to *P. aeruginosa* infection. (**a**) Representative images depicted lung sections (H&E) from control and groups of mice exposed to PAK for 0 (control), 24, 28, 72 and 96 h. Dashed boxes are enlarged and depicted below. Arrows indicate infiltrates and accumulation of cellular debris in alveolar space. (**b**) The lung injury score from indicated groups of mice. (**c**) Therapeutic interventions with metformin and Tideglusib initiated during established lung injury, i.e., 24 h after exposure mice to PAK. (**d-e**) Lung sections (H&E) and (**f**) the lung injury score for indicated groups of mice. Data presented as Box plots, *n* = 5 mice/group. *P < 0.05 (ANOVA)

**Fig. 2:**
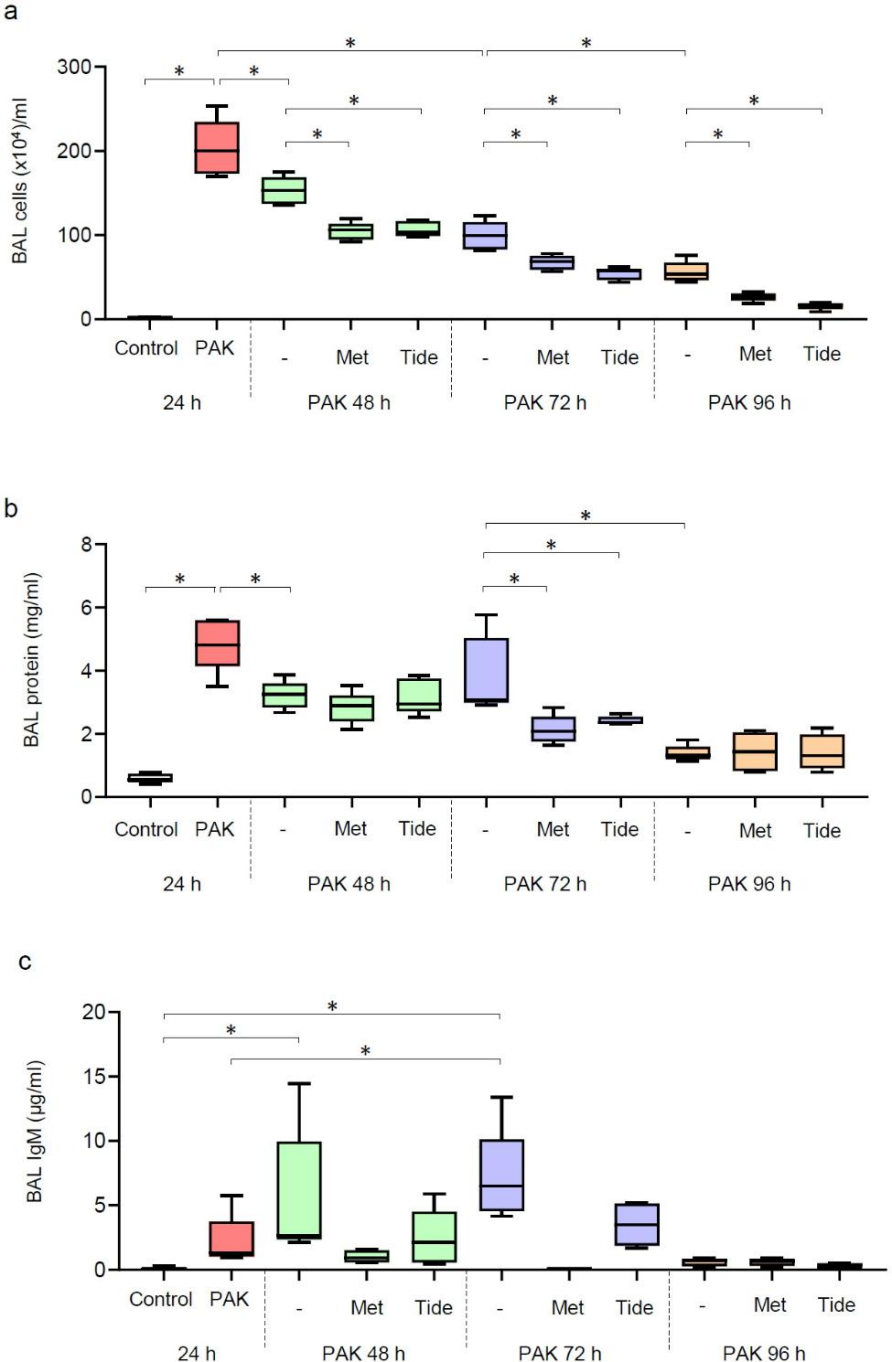
Metformin and Tideglusib reduced immune cell flux and diminished lung barrier permeability in mice subjected to PAK-induced lung injury. (**a**) BAL white cell, (**b**) BAL fluid protein content, and (**c**) IgM in BAL fluids from indicated groups of mice. Data presented as Box plots, with *n* = 5 mice/group. *P < 0.05 (ANOVA)

To investigate the process of resolution after bacteria-induced lung injury, mice received a significant, but non-lethal dose of wild-type strain K *P. aeruginosa* (PAK; 2.5 × 10^7^/50 µl PBS/mouse; orthopharyngeal administration). Lung sections and bronchoalveolar (BAL) fluids were processed from control and groups of mice subjected to PAK for 24, 48, 72 and 96 h. Acute lung injury (ALI) is evidenced by infiltration of inflammatory cells, in particular neutrophil influx and accumulation of cellular debris within alveolar spaces (Fig. 1a). This is associated with a thickened septum in mice subjected to PAK, as evidenced 24 h post infection (**Supplementary Fig. 1**). Notably, lung injury persists for 48 and 72 h and substantially decrease 96 h post-infection (Fig. 1b). Next, we tested the therapeutic potential of AMPK activators metformin and Tideglusib. Metformin is a commonly used drug for type 2 diabetes mellitus (T2DM); however, it indirectly activates AMPK, while Tideglusib is a potent, selective and irreversible non-ATP-competitive suppressor of the AMPK inhibitor GSK-3β, with previously established efficacy in animal models and in recent clinical trials for Alzheimer’s disease [40]. Mice received metformin (65 mg/kg; i.p.) or Tideglusib (50 mg/kg; i.p.) during an established pneumonia-induced ALI, i.e., 24 h after exposure to PAK. Both, metformin and Tideglusib effectively accelerated the recovery from lung injury, as evidenced by improved lung architecture, reduced amounts of neutrophil and proteinaceous material in alveolar space at the 48, 72 h, as compared to PAK alone (Fig. 1d-f and Fig. 2a). Of note, in mice that received PAK for 24 h, there was a significant accumulation of white cells in BAL fluids. BAL fluid cell count was reduced at 48, 72, and 96 h time points (Fig. 2a). This recovery is associated with a significant decrease in barrier permeability, indicated by reduced protein content and IgM in BAL fluids in either metformin or Tideglusib-treated mice, in particular 72 h post PAK injection (Fig. 2b,c). Notably, at the tested dosages, the enhanced permeability and accumulation of inflammatory cells mostly subsided in all groups of mice, including mice exposed to PAK alone for 96 h (Fig. 2b).

### Metformin and Tideglusib-dependent recovery of AMPK activity during pneumonia- established lung injury

**Fig. 3:**
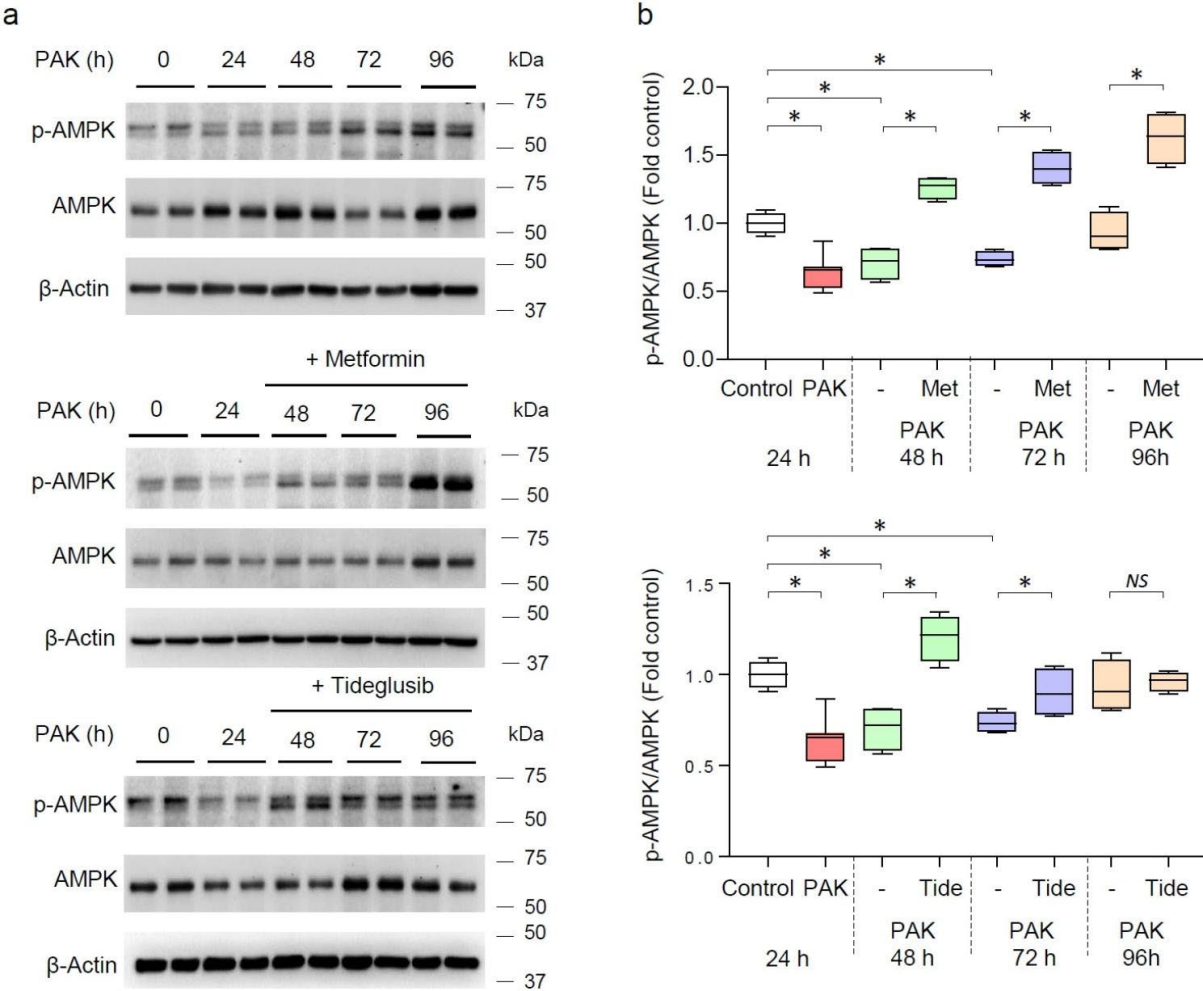
Metformin and Tideglusib-dependent recovery of AMPK phosphorylation (activity) in lungs of mice subjected to PAK-induced lung injury. (**a**) Representative western blots and (**b**) quantitative analysis of AMPK phosphorylation (pThr172-AMPK) and total amounts of AMPK and β-actin in lung homogenates from indicated groups of mice. Immunoblots consist 2 samples from individual lung homogenates per group, while quantitative data included *n* = 5 per indicated groups of mice. Data presented as Box plots, fold changes of control (untreated), *P < 0.05 (ANOVA)

AMPK phosphorylation is significantly reduced in mice treated with PAK for 24, 48, and 72 h, as compared to control (untreated) mice (Fig. 3a-b). Metformin and Tideglusib effectively increased AMPK phosphorylation at the indicated time-points post-PAK treatment. Notably, metformin was more effective as compared to Tideglusib, likely due to the stimulatory effects of metformin *versus* the ability of Tideglusib to prevent AMPK de-phosphorylation.

### Metformin and Tideglusib promote recovery from ER stress in alveolar epithelial cells after PAK-induce lung injury

**Fig. 4:**
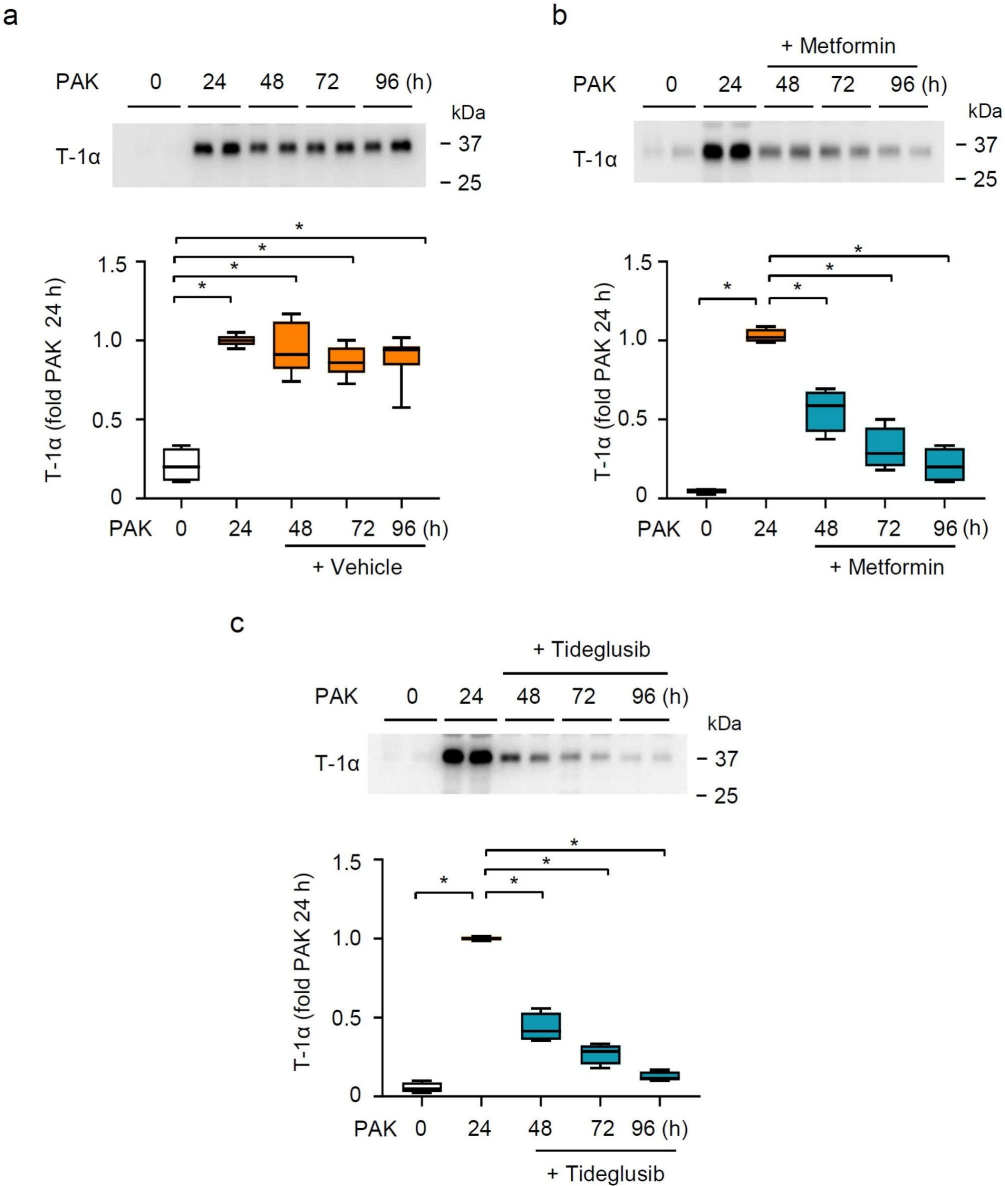
AMPK activation reduces the accumulation of AEC surface marker T-1α in BAL fluids of PAK-treated mice. (**a-c**) Representative immunoblots and quantitative analysis of T-1α in BAL fluids from control and mice treated with (**a**) PAK alone or PAK and subsequent treatment with (**b**) metformin or (**c**) Tideglusib. Representative immunoblots contain 2 samples of BAL fluids from individual mice/group, while quantitative analysis included samples from 5 mice/group. Data presented as fold changes of T-1α in PAK-treated mice for 24 h. Box plots are showed, *P < 0.05 (ANOVA)

Exposure to PAK resulted in significant and persistent (i.e., 24–96 h time period) damage of AECs, as indicated by the accumulation of alveolar epithelial type I cell surface membrane marker T-1α in the BAL fluids (Fig. 4a). The analysis of BALFs for T-1α was performed after BALFs were centrifuge to remove insoluble fractions, likely to contain cells and cellular debris. Of note, while T-1α is predominantly expressed marker in AECs, which can be released into the alveolar space upon death, it is possible that dying macrophages may also release T-1α [41]. This result demonstrates a prolonged injury of alveolar epithelium after PAK instillation. Notably, such injury persisted despite decrease in initial inflammatory responses (**Supplementary Fig. 2**). In regards to AECs injury, metformin or Tideglusib effectively reduced and further nearly completely normalized the levels of T-1α in the BAL fluids, as early as administration of AMPK activators for 24 h, compared to groups of mice subjected to PAK alone (Fig. 4b,c).

**Fig. 5:**
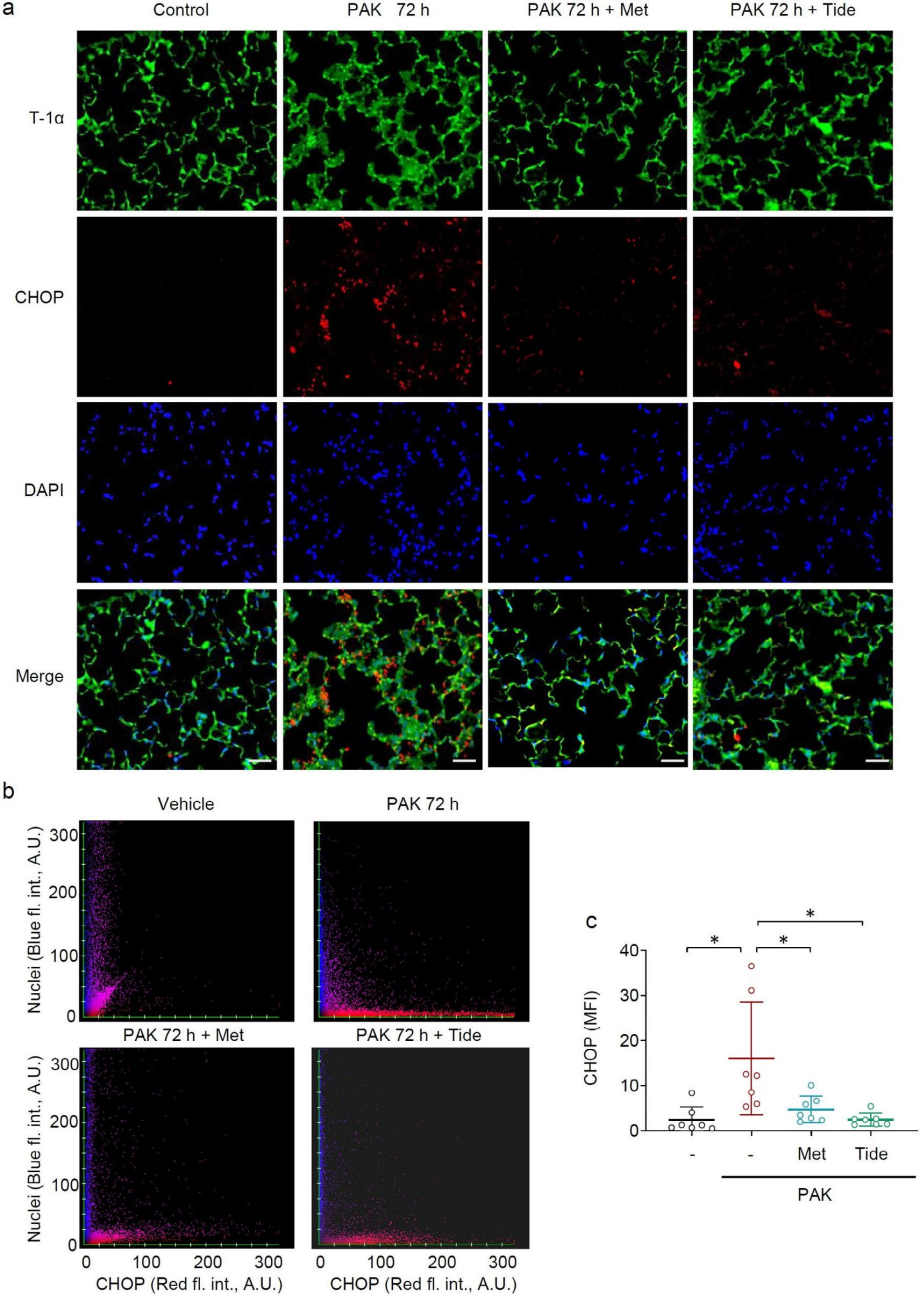
PAK-induces ER-stress in AECs is reduced upon therapeutic administration of AMPK activators. (**a**) Representative images show fluorescence patterns of CHOP (red), T-1α (green; AECs Type 1 marker) and nuclei (blue) in lung sections from control and mice exposed to PAK for 72 h. Metformin (Met) or Tideglusib (Tide) were administered 24 h post PAK infection and lung sections obtained 48 h afterwards. Scale bar 100 μm. (**b**) Scattergrams indicate correlation of nuclei/CHOP fluorescence intensity (A.U.; arbitrary units) for corresponding images in (a). (**c**) Quantitative analysis of CHOP fluorescence intensity in lung sections from indicated groups of mice. Data presented as Scattered plot with fold changes of control (untreated) mice, mean ± s.d., *n* = 7 images/per indicated groups of mice. *P < 0.05 (ANOVA)

**Fig. 6:**
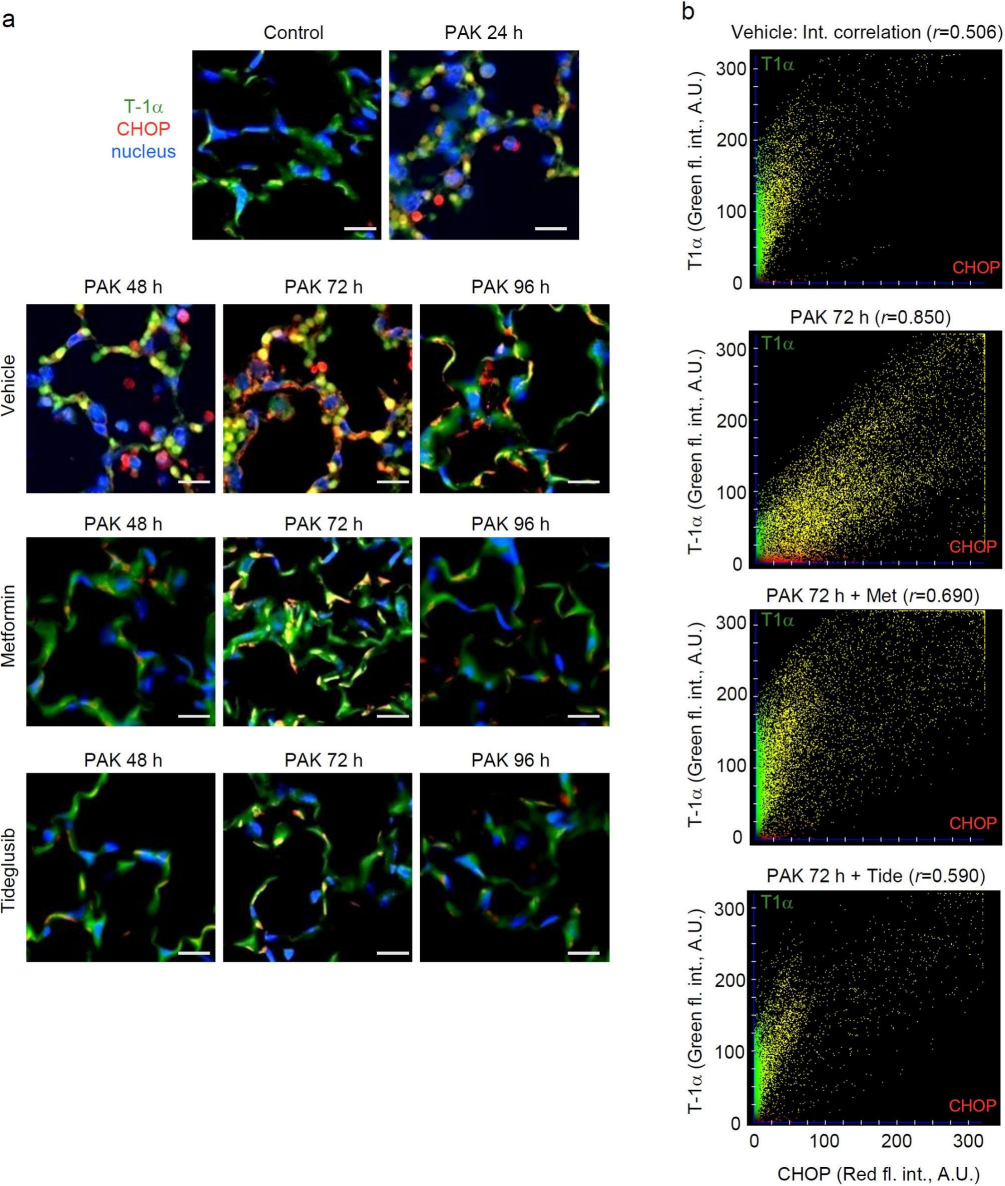
Metformin and Tideglusib decrease of ER-stress marker CHOP in AECs of PAK-infected mice. (**a**) Representative images and (**b**) fluorescence intensity scattergrams showed patterns of CHOP (red) accumulation, T-1α AECs (Type 1) fluorescence (green) and nuclei (blue) in lung sections from control and mice exposed to PAK alone for 24, 48, 72 and 96 h. Mice received metformin or Tideglusib 24 h post PAK-induced lung injury. Scattergrams indicate correlation of T-1α/CHOP fluorescence intensity (A.U.; arbitrary units)

**Fig. 7:**
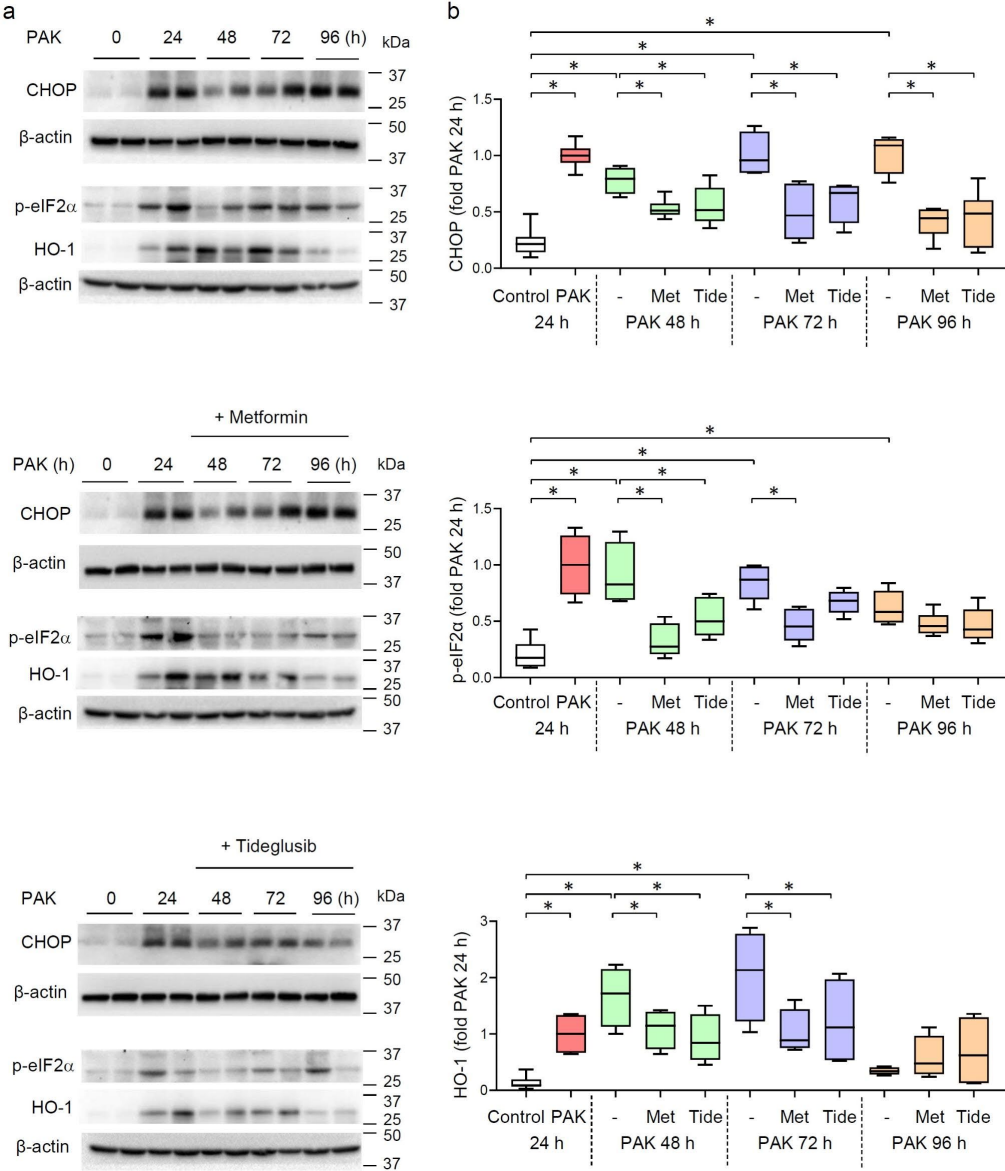
Metformin and Tideglusib reduced CHOP accumulation in lung homogenates from mice with PAK-induced lung injury. (**a**) Representative immunoblots and (**b**) optical bends densitometry of CHOP, p-eIF2α, HO-1 and β-actin (loading control) were from lung homogenates of control, mice treated with PAK alone and PAK followed by treatment with metformin or Tideglusib. Immunoblots consist samples from two lung homogenates/group, while quantitative data included samples from *n* = 5 mice/group. Data presented as Box plots with fold changes of control (untreated), *P < 0.05 (ANOVA)

ER stress is typically associated with the accumulation of CCAAT/enhancer-binding protein-homologous protein (CHOP). In particular, the most pronounced accumulation of CHOP was evidenced 72 h after PAK-induced lung injury (Fig. 5). Metformin and Tideglusib reduced the number of cells positive for CHOP, as depicted by co-immunofluorescence staining CHOP and alveolar epithelial cells (Type I) in lung sections from mice treated with PAK for 72 h (Fig. 6a,b). To further investigate the effects of AMPK activators on ER-stress, CHOP is measured in whole lung homogenates. This assay is not exclusive for AECs, although important given nearly 70% of lung cells are AECs. We found that metformin and Tideglusib have a significant impact on ER stress (Fig. 7), in particular CHOP, eIF-α and HO-1 levels are diminished after exposure to AMPK activators for 24, 48, 72 and 96 h post PAK (Fig. 7a,b).

### AMPK activation reduces AECs apoptosis and promotes clearance of apoptotic cells in lung of mice after ***P. aeruginosa***-induced lung injury

**Fig. 8:**
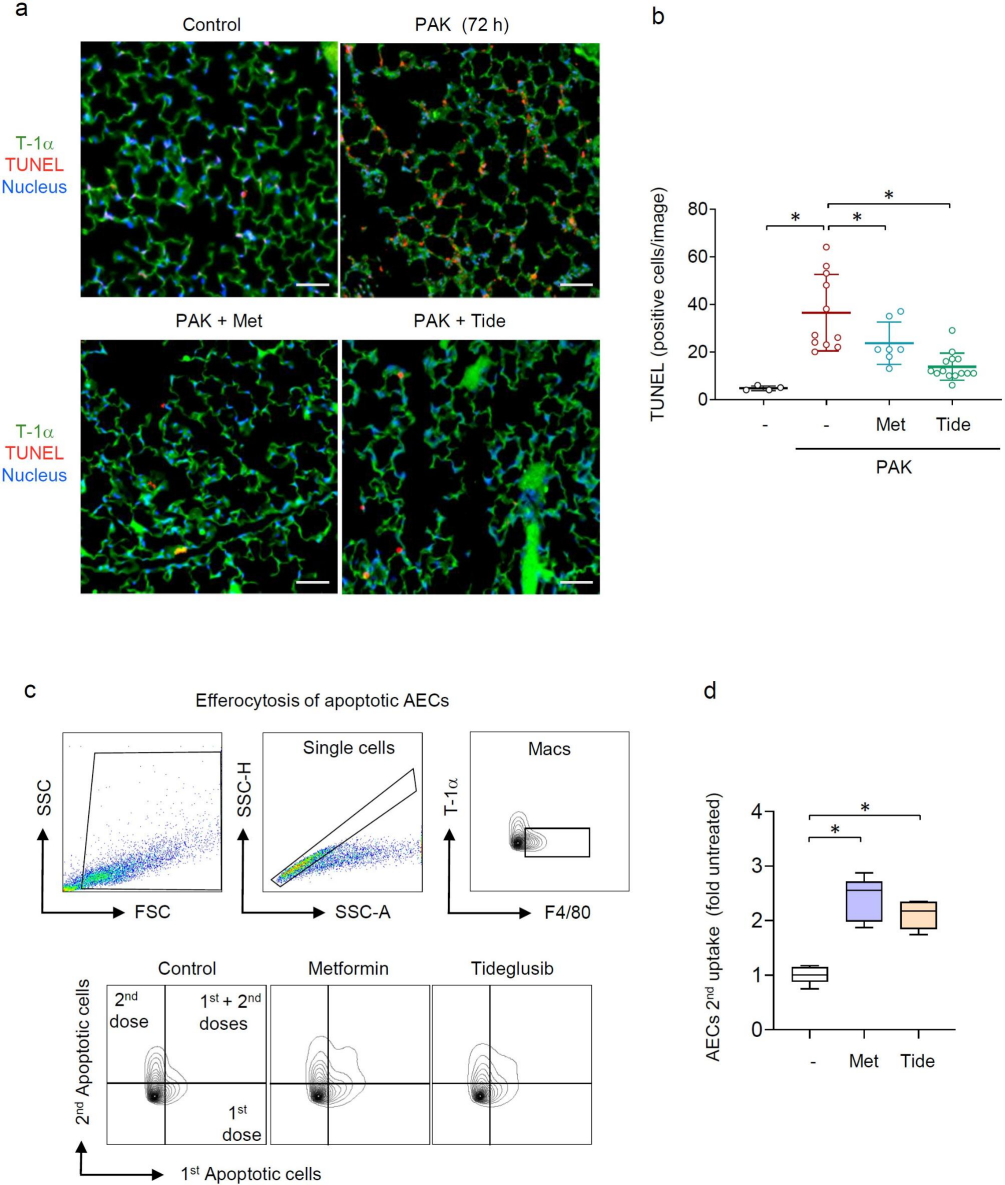
Delayed activation of AMPK reduces AECs apoptosis in PAK-infected mice. (**a**) Representative images depicted fluorescence patterns of apoptotic cells (TUNEL) and AECs (T-1α) in the lung sections from indicted groups of mice, T-1α (green), TUNEL (red), nucleus (blue). Scale bar 100 μm. (**b**) Quantitative analysis of apoptotic cells. Scatter dot plot with data presented as fold control (untreated), mean ± s.d., *n* = 4–14 images/per indicated groups of mice. *P < 0.05 (ANOVA). (**c,d**) The effects of metformin and Tideglusib on macrophage-dependent continuous clearance of apoptotic AECs. (**c**) Representative flow cytometry charts, and (**d**) quantitative analysis of apoptotic AECs uptake by macrophages, ex vivo. Macrophages received dose one of apoptotic cells for 4 h and then a second dose for an additional 4 h. Representative flow charts (gating strategy) are showed along with Box plot. Data are presented as fold changes of control for *n* = 6 independent samples/group. *P < 0.05 (ANOVA)

Given that lung injury is associated with ER stress-related cell death, we investigated if AMPK activators affect AECs apoptosis. At the time of established ER-stress, i.e., 72 h post exposure to PAK, there is a significant accumulation of apoptotic alveolar cells and other cell populations, as indicated by fluorescence images of (TUNEL staining) from lung sections (Fig. 8a). In this experiment, we performed the triple staining for T-1α (green); TUNEL (red), and nuclei (blue) to indicate a number of apoptotic cells in lung sections. Although orange fluorescence is a resultant of overlap between the T-1α (green) and TUNEL (red), we also found red positive cells that do not have the epithelial marker, indicate that other cell populations undergo apoptosis after exposure to PAK. Importantly, metformin or Tideglusib effectively diminished the abundance of apoptotic cells (Fig. 8a,b). It is important to note that the accumulation of apoptotic cells is not only dependent on the rate of dying cells, but also affected by the ability of lung macrophages to neutralize apoptotic cells, also known as efferocytosis [42, 43]. Given that efferocytosis plays a crucial role in resolution from inflammatory conditions and wound healing, clearance of apoptotic cells was measured upon exposure of peritoneal mature macrophages to metformin (0 or 0.5 mM) and Tideglusib (0 or 30 µM) for 18 h. The efferocytosis assay was conducted by inclusion of the first dose of apoptotic alveolar epithelial cells (primary murine AECs Type I) labeled with PKH-26 fluorescent dye for 2 h. Next, the second dose of apoptotic AECs-PKH-67 were included for an additional 45 min followed by flow cytometry of macrophage with lineage marker T-1α. Metformin and Tideglusib preserved a continuous clearance of apoptotic AECs (Fig. 8c,d). These results indicate that along with a reduced ER-stress in AECs, the number of apoptotic cells can also be affected by AMPK-dependent enhancement of efferocytosis.

## Discussion

This is the first study, to our knowledge, to demonstrate the therapeutic potential of AMPK activators to reduce ER-stress in AECs and related lung injury in an experimental model of pneumonia. Exposure to PAK resulted in the development of lung injury that is characterized by neutrophil accumulation, enhanced alveolar permeability, localized alveolar damage and apoptosis of AECs. We found that therapeutic interventions with metformin and Tideglusib led to activation of AMPK followed by a significant decrease of ER-stress and related apoptosis of AECs post-PAK groups of mice. In particular, the effects of AMPK activators are correlated with the reduction in the ER-stress marker CHOP and diminished accumulation of the AEC cell surface marker T-1α in BAL fluids, indicative of recovery/preservation of AECs viability. Notably, while the benefits of AMPK activators are attributed to reduced ER-stress and apoptosis, it is possible that recovery from pneumonia is also mediated by enhanced clearance of apoptotic cells. Indeed, metformin or Tideglusib preserved macrophage-dependent continuous uptake/neutralization of apoptotic AECs, ex vivo. Collectively, our studies indicate that recovery from bacterial pneumonia can be accelerated by restoration of AMPK activity.

ER-stress is closely linked to bioenergetic and metabolic alterations caused by bacterial pathogens and inflammatory lung injury. Our study provides several important insights into AMPK-dependent regulation of AEC viability during lung infection-related ALI. Previous studies, including our own, described the pre-emptive effects of AMPK on mitochondrial function in ALI [20, 22, 23, 44]. Our current study suggests that therapeutic interventions targeting AMPK activation can also improve mitochondria-ER crosstalk, thereby preserving AEC viability that declines significantly over 3–5 days post infection. This is a plausible concept, given that the ER and mitochondria are joined together at multiple contact sites, referred to as mitochondria-ER associated membranes (MAMs) [45]. Although mitochondria and ER have distinct biochemical properties, they communicate *via* the MAM interface which facilitates efficient regulation of energy production, lipid processing, Ca^2+^ buffering/signaling, and cell viability. In this setting, AMPK activators are known to improve mitochondrial function; this important therapeutic interventions counter inflammation-dependent impairment of AMPK and mitochondrial bioenergetics. Loss of AMPK activity and mitochondria homeostasis is likely affecting ER stress, leading to exacerbated perturbations in protein trafficking and accumulation of misfolded/unfolded proteins. Notably, ER stress and loss of proteostasis have been implicated in various inflammatory conditions associated with cardiovascular and neurodegenerative disorders. In metabolic syndromes including in diabetes and obesity, ER stress is an accepted contributor to pathologies defined by metabolic/bioenergetic impairment, chronic low-grade inflammation and insulin resistance. In this context, AMPK activation produces a beneficial outcome through reduction of LDL-induced ER-stress [46]. Metformin is also capable of reducing palmitate-induced ER stress in liver cells [47]. A more direct link between mitochondria and ER-stress is supported by observations in pancreatic B-cells that AMPK phosphorylation of Drp1 improves endothelial function by suppressing mitochondrial-ROS-associated ER stress in the endothelium and ER morphology [48, 49]. We have recently reported that AMPK activation by metformin also reduced ER-stress and related emphysema in patients with COPD and in a murine model of cigarette smoke and aging [50].

Although endotoxin challenge has been shown to increase ER stress markers in the murine lungs, the hypothesis that therapeutic targeting of bioenergetic impairment could impact ER-stress during severe lung infections has not been previously tested. We demonstrate that both metformin and Tideglusib effectively reduced CHOP in the lungs of mice subjected to PAK-induced ALI. Our findings are significant, given that failure to resolve ER stress *via* adaptive UPR signaling is closely followed by induction of ER stress-dependent apoptotic signaling pathways, such as CHOP. Another possible mechanism by which of AMPK affects ER-stress is suppression of glucose-regulated protein 78 (GRP78), an ER chaperone that confers protection against stressors by mediating protein refolding. A recent study has indicated that AMPK reduces GRP78 expression and increases heme oxygenase-1 expression in a mouse model of ER stress-induced kidney injury and fibrosis [29]. Less is known about the impact of AMPK on ER-stress associated with lung injury, though AMPK is capable of regulating ER stress by inducing the ER-chaperone ORP150 [51]. Notably, the potential effects of AMPK on ER proteins degraded by ERAD and/or autophagy are supporting the notion that AMPK-autophagy axis is as important as ERAD for degradation of terminally misfolded ER proteins [52]. It is important to note about the complex role of AMPK that plays in repair and resolution of lung injury. In particular, AMPK activators emerged as a novel approach that mediates anti-fibrotic effects *via* multiple pro-resolution mechanisms [26, 27, 53]. AMPK also supports clearance of apoptotic cells. In particular, while the initial response to bacterial infection or apoptotic cells is known to activate phagocytosis, this capacity is reduced or completely diminished in time. Although the mechanisms involved in defective efferocytosis are not well understood, several studies emphasized that loss of clearance of apoptotic cells is an important issue associated with several lung diseases [54].

*P. aeruginosa* is a formidable opportunistic and antibiotic resistant pathogen, leading to chronic and highly lethal infections in immune compromised hosts [55–61]. Bacterial pneumonia is the most common lower respiratory infection induced by *P. aeruginosa* and the leading cause of acute nosocomial infections. In particular, ventilator-associated pneumonia (VAP) is a significant cause of morbidity and mortality in critically ill patients, and the isolation of *P. aeruginosa* is associated with worse clinical outcomes [62, 63]. It is important to note that while *P. aeruginosa* is among the most frequent cause of pneumonia in ICU, other microbial pathogens contribute to nosocomial infection and the development of lung injury. In addition, lung infections and AEC apoptosis may be closely linked to progressive and irreversible lung remodeling which may ultimately leads to lung fibrosis. However, it is unclear precisely in which cell types ER stress plays a role in the pathogenesis of pulmonary fibrosis [14, 15]. In ARDS and in experimental models of ALI, pathogenic bacteria and inflammatory conditions are implicated in alveolar epithelial damage and the activation of cell death programs. The apoptotic cell accumulation is not only dependent on the loss of cell viability, but also the rate of apoptotic cell clearance [42, 64]. While efferocytosis plays crucial roles in the organ development and tissue homeostasis, less is known about mechanisms and outcomes of efferocytosis inhibition in acute conditions of lung injury and chronic lung disorders [65]. Given that the apoptotic cell accumulation is not only a result of apoptosis, we also explored a possibility that AMPK activation can support clearance of apoptotic cells, in particular, macrophage-dependent efferocytosis performance. Our findings suggest that accumulation of apoptotic cells is affected by both, AMPK-dependent improvement of AECs viability and the AMPK stimulated continuous clearance of apoptotic cells by macrophages, as evidenced ex vivo. Thus, AMPK may have synergistic therapeutic effects *via* protection of AECs and clearance of apoptotic cells. However, despite positive outcomes of metformin in several models of sepsis [19, 20, 22, 25, 66–68], metformin is not administered in ICU patients due to a potential risk of causing or worsening lactic acidosis [69, 70]. It is important to note that our study indicates a possibility for treatment with metformin or Tideglusib during post-septic conditions. In particular, future metformin interventional studies may be justified to accelerate the recovery from lung injury. But in such studies, the initiation of metformin administration may only begin after critically ill patients’ lactate levels have clearly normalized.

## Conclusion

In summary, AMPK activation is an essential target not only in the context of impaired cellular bioenergetics, but also to alleviate ER-stress during inflammatory conditions and related bacterial lung infections. While ER stress is associated with a prolonged lung injury and apoptosis of AECs, our studies highlight the ability of AMPK activators to accelerate the recovery after bacterial infections.

## Electronic supplementary material

Below is the link to the electronic supplementary material.

Supplementary Material 1

## Data Availability

Data supporting the findings of this study are available from the corresponding author upon request.

## List of abbreviations

AMPK: AMP-activated protein kinase
ADP: Adenosine diphosphate
AEC: Alveolar epithelial cell
ALI: Acute lung injury
AMP: Adenosine monophosphate
ARDS: Acute respiratory distress syndrome
ATP: Adenosine triphosphate
BAL: Bronchoalveolar lavage
CHOP: CCAAT/enhancer-binding protein-homologous protein
COPD: Chronic obstructive pulmonary disease
DAPI: 4′,6-diamidino-2-phenylindole
Drp1: Modulation of Dynamin-related Protein 1
ER: Endoplasmic reticulum
eIF-α: Eukaryotic translation elongation factor alpha
GRP78: Glucose-regulated protein 78
GSK-3β: Glycogen synthase kinase 3β
H&E: Hematoxylin and eosin
HO-1: Heme oxygenase 1
ICU: Intensive care unit
LDL: Low-density lipoprotein
MAMs: Mitochondria-ER associated membranes
ORP150: ER-chaperone, 150-kDa oxygen-regulated protein
PAK: P. aeruginosa, wild-type strain K
PBS: Phosphate buffered saline
T-1a: AEC Type 1 cell surface marker
TUNEL: Terminal deoxynucleotidyl transferase dUTP nick end labeling
UPR: Unfolded protein response
VAP: Ventilator-associated pneumonia
