# Supplementary material for: AMPK activation improves recovery from pneumonia-induced lung injury via reduction of er-stress and apoptosis in alveolar epithelial cells
Source: Respir Res. 2023 Jul 12;24:185. doi: 10.1186/s12931-023-02483-6 (PMC10337128; doi:10.1186/s12931-023-02483-6)
Supplement: Supplementary file 1 — Supplementary Material 1 [file 12931_2023_2483_MOESM1_ESM.pdf]

Supplementary Figure 1

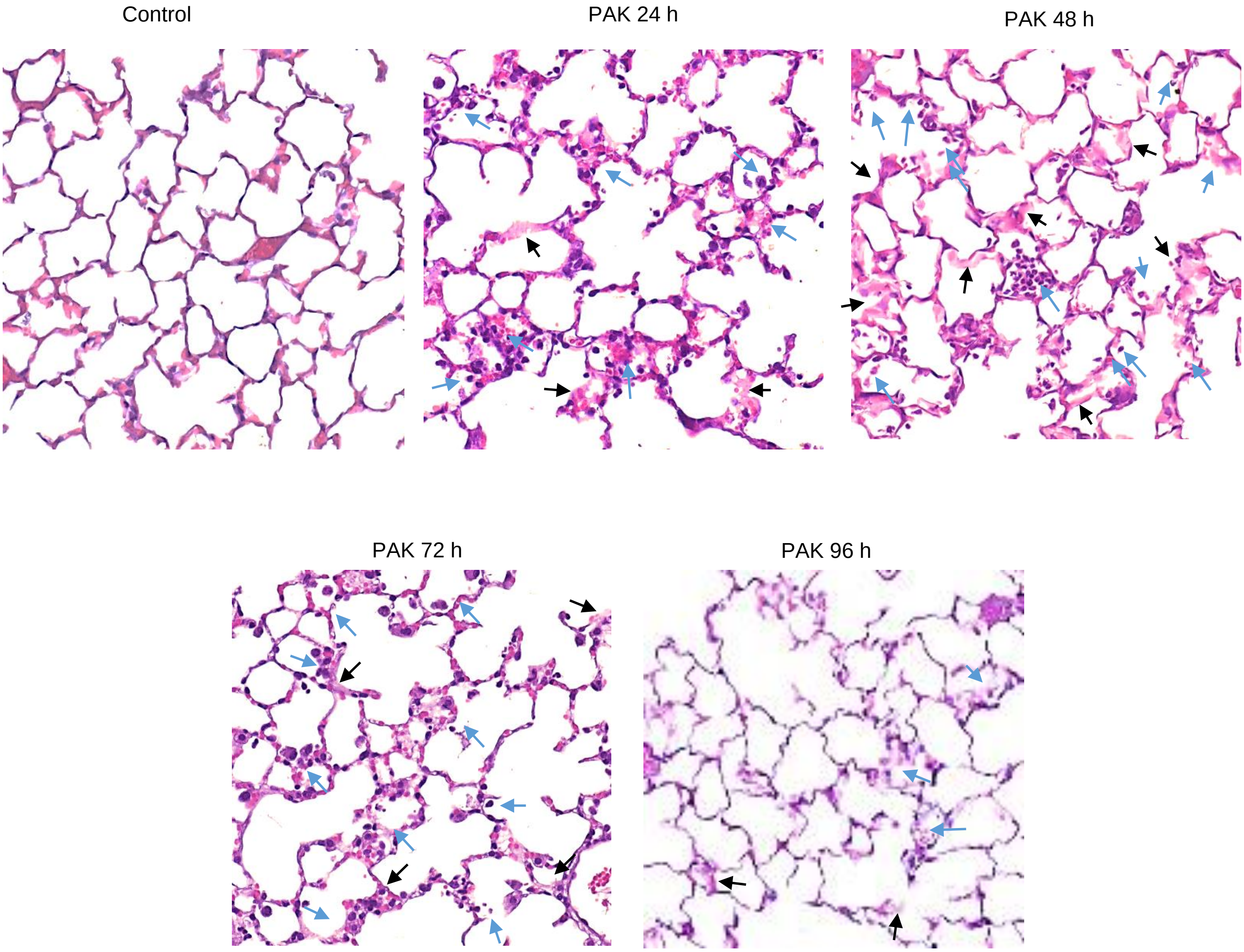

Supplementary Figure 1. **AMPK activation improved recovery from *P. aeruginosa*-induced lung injury.** (**a**) Representative images depicted lung sections (H&E) from control and groups of mice exposed to PAK for 0 (control), 24, 28, 72 and 96 hours. Arrows (blue) showed neutrophil flux and accumulation of cellular debris, as well as thickened septum (black arrows).

Supplementary Fig. 2

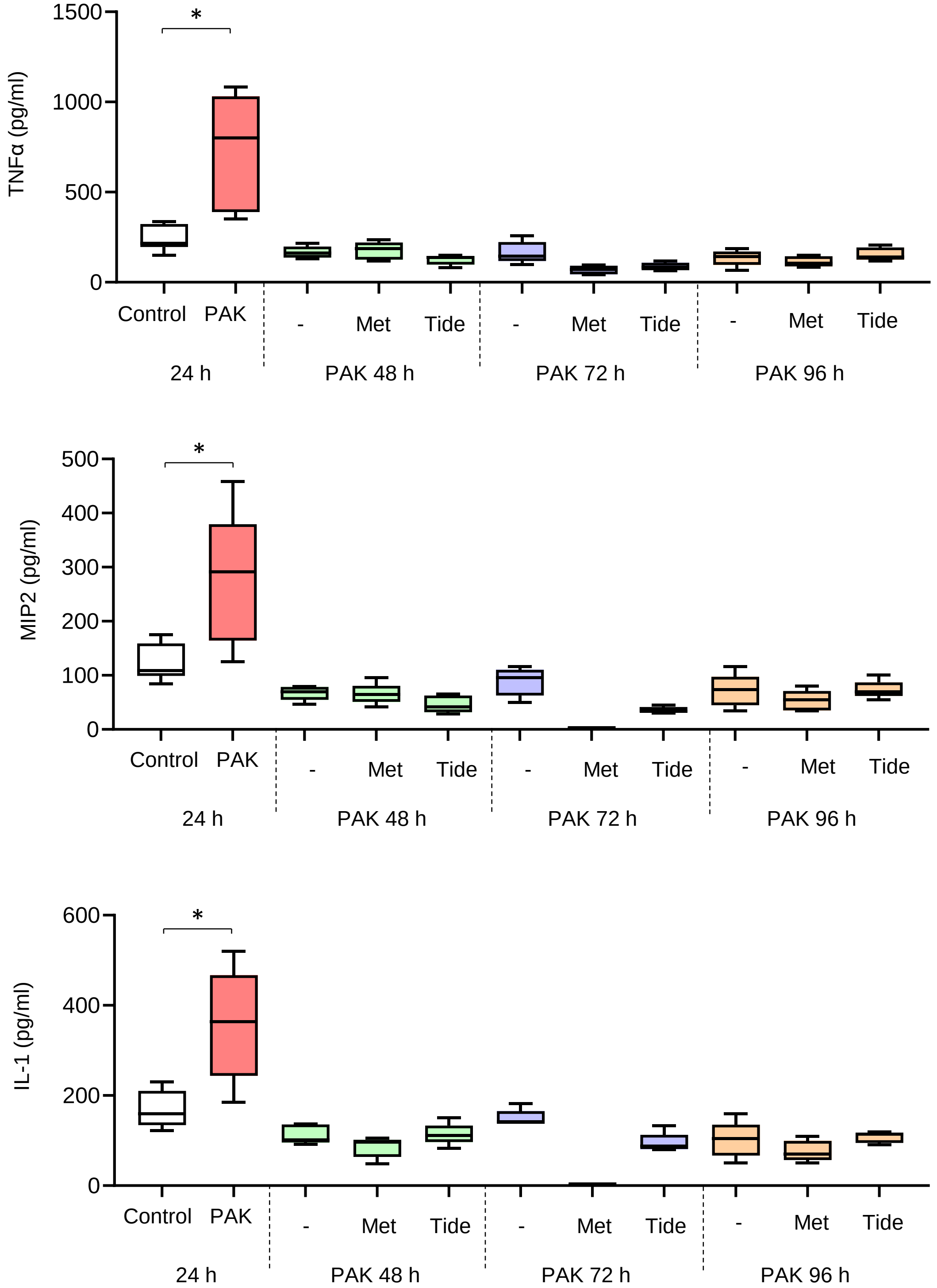

Supplementary Fig. 2. The amounts of TNF-α, MIP2 and IL-1 in BAL fluids from control, PAK or PAK and metformin or Tideglusib-treated mice. Data presented as Box plot, mean ± s.d. from *n* = 5 mice/ group.**P*<0.05 (ANOVA)
